# Tick-borne encephalitis foci in northeast Italy revealed by combined virus detection in ticks, serosurvey on goats and human cases

**DOI:** 10.1080/22221751.2020.1730246

**Published:** 2020-02-26

**Authors:** Niccolò Alfano, Valentina Tagliapietra, Fausta Rosso, Ute Ziegler, Daniele Arnoldi, Annapaola Rizzoli

**Affiliations:** a Fondazione Edmund Mach, Research and Innovation Centre, Trento, Italy; b Friederich-Loeffler-Institute, Federal Research Institute for Animal Health, Institute of Novel and Emerging Infectious Diseases, Greifswald-Insel Riems, Germany

**Keywords:** Tick-borne encephalitis, prevalence, *Ixodes ricinus*, goats, Italy, human cases, phylogenetic analysis

## Abstract

Tick-borne encephalitis (TBE) is a severe zoonotic neurological disease endemic in northeast Italy since 1992. In the Province of Trento, a sharp increase in TBE incidence has been recorded since 2012, despite the vaccination efforts. To assess current TBE infection hazard in this area, we applied an integrated approach combining the distribution of human cases, the seroprevalence of tick-borne encephalitis virus (TBEV) in sentinel hosts and the screening of questing ticks for TBEV. A total of 706 goat sera from 69 farms were screened for TBEV-specific antibodies resulting in 5 positive farms, while the location of human cases was provided by the local Public Health Agency. Tick sampling was concentrated in areas where TBEV circulation was suggested by either seroprevalence in goats or human cases, resulting in 2,410 *Ixodes ricinus* collected and analyzed by real-time RT–PCR. Four tick samples from 2 areas with record of human cases were positive to TBEV corresponding to a 0.17% prevalence in the region, while risk areas suggested by serology on goats were not confirmed by tick screening. Our results revealed an increase in TBEV prevalence in ticks and the emergence of new active TBE foci, compared to previous surveys, and demonstrated the importance of an integrated approach for TBE risk assessment. A phylogenetic analysis of the partial E gene confirmed that the European TBEV subtype is circulating in northeast Italy and suggested that the different Italian TBEV strains originated independently as a result of different introductions from neighbouring countries, presumably through migratory birds.

## Introduction

Tick-borne encephalitis (TBE) is an emerging tick-borne zoonosis characterized by severe acute and chronic neurological infections. It is caused by a single-stranded RNA virus belonging to the mammalian tick-borne virus group within the family *Flaviviridae* [1]. The tick-borne encephalitis virus (TBEV) consists of three main subtypes: European (TBEV-Eu), Siberian (TBEV-Sib), and Far Eastern (TBEV-FE), which are characterized by different vector competence, geographical distribution throughout Europe and Asia, and pathogenicity to humans [2]. Recently, two additional subtypes, Baikalian (TBEV-Bkl) and Himalayan (TBEV-Him), have been identified [3,4]. TBEV-Eu, which is the least virulent subtype (mortality rate <2%) [5], is endemic in rural and forested areas of central, eastern and northern Europe and has *Ixodes ricinus* as its main tick vector.

Within its geographical range TBEV shows a patchy distribution in small natural foci [6,7], where it circulates between tick vectors and vertebrate hosts. Among these, rodents and insectivores act both as reservoirs and amplifier hosts for the virus [8], with ticks which may become infected either by feeding on a viraemic host or, more efficiently, by co-feeding, typically at the nymph or larval stage, next to an infected tick on a non-viraemic host [9]. Other species, like goats, sheeps and cattle, do not develop sufficient viraemia to support direct viral transmission to ticks [10], but can excrete the virus through milk causing alimentary TBE infections in humans due to the consumption of unpasteurized milk or dairy products [6]. In addition, they develop an immune response with production of persistent TBEV-specific antibodies, and thus can serve as sentinel hosts for serological monitoring [8,11,12].

More than 10,000 cases of TBE arise every year in Eurasia, with about 3,000 reported in Europe [13,14], where TBE is endemic in 27 countries and is a notifiable disease since 2012. During the last decades, TBE has expanded northward and at higher altitudes, new endemic foci have been discovered and an increase in cases has been registered in many European countries [14]. Several factors can explain this trend, such as the effect of climate change on vector and host distribution, increased outdoor recreational human activities, an higher public awareness and the widespread application of diagnostic methods [15,16].

Italy, where TBE has been reported since 1967 and the virus was first isolated in 1975 [17], is considered a country at low risk for TBE, which seems to be restricted to a relatively small fraction of its territory, corresponding to pre-alpine and alpine regions in the northeastern part of the country (“Triveneto” area): Trentino-Alto Adige, Veneto and Friuli Venezia Giulia [18]. In this area the number of cases has sensibly increased over time, with 131 cases diagnosed during the period 1975–2004 (4.5 cases/year) [19] and 367 between 2000 and 2013 (28 cases/year) [18], and the mean annual incidence (number of cases per 100,000 inhabitants) of TBE increasing from 0.06 in 1992 to 0.88 in 2006 [20]. Therefore, Triveneto is considered an area historically endemic for TBE. The Province of Trento (part of the Trentino-Alto Adige region), where TBE had been registered with low incidence (0.6) between 1992 and 2004 [19], was recently identified as one of the three provinces of Triveneto with the highest TBE incidence [18]. The last surveys of TBEV prevalence in tick population in the Province of Trento date back to 1996–1997 [21] and 2006 [22], when strains of TBEV-Eu were found circulating in the area. However, the results of these studies are outdated or restricted to a very limited area of this territory [22]. Aim of this study was to identify active TBEV foci and assess current TBE hazard in the Province of Trento, by performing a wide serological screening of TBEV in vertebrate sentinel hosts (goats) and determining the virus prevalence in questing ticks collected from a wide area of this territory. We concentrated tick sampling in areas suspected of TBEV circulation by other evidences, either where goats were found seropositive to TBEV or the most recent human TBE cases were reported. At the same time, we aimed at characterizing the TBEV strains circulating in the area by molecular and phylogenetic analysis.

## Materials and methods

### Human TBE cases

**Figure 1.**
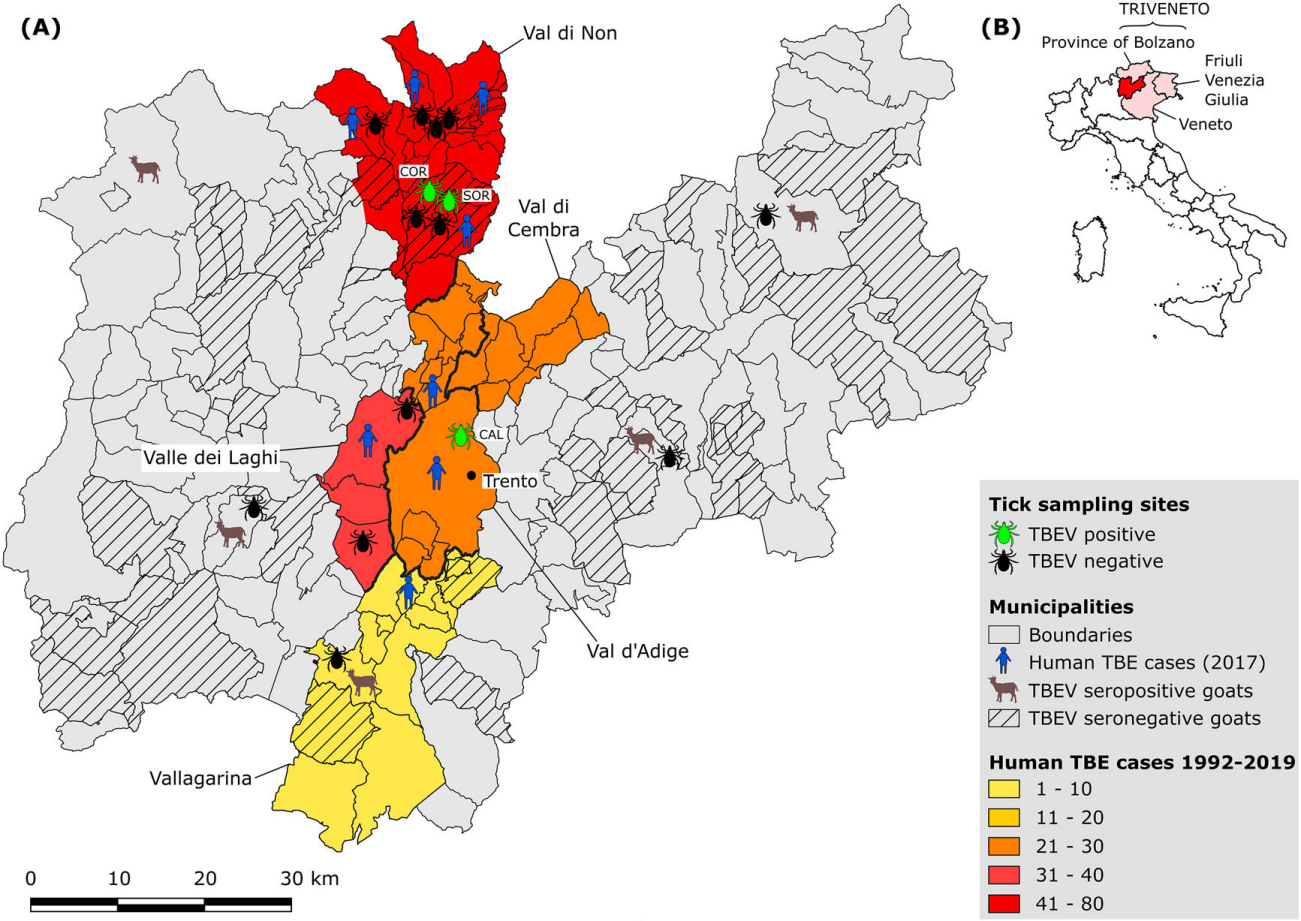
(A) Map of the Province of Trento reporting the locations of (i) the tick sampling sites; (ii) of the farms providing goat serum samples; (iii) of the human TBE cases. Thicker lines mark the borders between valleys. (B) Map of Italy showing the location of the Province of Trento within Triveneto.

**Figure 2.**
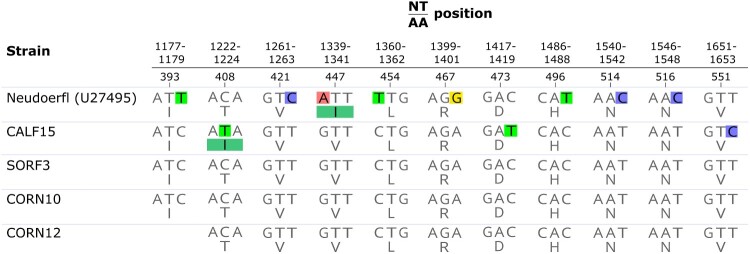
Human TBE cases reported by valley in the Province of Trento from 1992 to 2019. The incidence rate (IR) was calculated as the number of cases/resident population per 100,000 inhabitants.

The number and the distribution of human clinical cases of TBE in the Province of Trento from 1992 to 2019 (Suppl. Tab. 1; Fig. 1; Fig. 2) and the localization by municipality of the cases reported in 2017 (Suppl. Tab. 2; Fig. 1) was provided by the local Public Health Agency (Azienda Provinciale per i Servizi Sanitari Provincia Autonoma di Trento, APSS).

### Collection of goat serum samples and TBEV serology

During 2017, a random subsample of 706 goat serum samples from 69 farms collected in the Province of Trento by the local Veterinary Hygiene and Public Health Agency during the ordinary surveillance activities was provided to our laboratory for TBEV screening (Suppl. Tab. 3). The municipalities corresponding to the sampled farms are shown in Figure 1. The serum samples were tested by ELISA for TBEV antibodies using the EIA TBEV Ig kit (TestLine Clinical Diagnostics) following manufacturer’s protocol. Since the standard TBEV-ELISA test commonly used on sheep and goat sera with species-specific cutoffs [12,23,24] is no longer available, and the other available kits designed for all vertebrate species have not been yet evaluated sufficiently on each species, the rate of false positive and borderline ELISA results are expected to be high and ELISA positive results need to be always verified by virus neutralization test (VNT). Therefore, all positive and borderline serum samples were tested for confirmation by VNT at the national reference laboratory for West Nile virus, Friederich-Loeffler-Institute, Greifswald, Germany. The VNTs were performed as already described [25,26] with the exclusion of serological cross-reactivities with other flaviviruses (WNV/USUV). Serum samples with ND50 ≥ 10 were evaluated as positive. Only samples confirmed by VNT were considered positive.

### Tick sampling and processing

Sixteen sampling sites dislocated across 7 valleys of the Province of Trento (Val di Non, Valle dei Laghi, Val d’Adige, Vallagarina, Valsugana, Valli Giudicarie, Val di Fiemme) (Table 1; Figure 1) were selected. Twelve sites were chosen according to the localization of human TBE cases reported in 2017 by the local Public Health Agency (Suppl. Tab. 2) and four according to the results of the serological screening on goats (Suppl. Tab. 3).

**Table 1.** *Ixodes ricinus* tick samples and TBEV prevalences in the Province of Trento.

							TBEV +			Prevalence by SITE			Prevalence by VALLEY			OVERALL Prevalence		
Site	Valley	Evidence	*N*	♂	♀	TOT	*N*	♂	♀	*N*	*A*	TOT	*N*	*A*	TOT	*N*	*A*	TOT
Smarano	Val di Non	human	109	8	6	123												
Tres	Val di Non	human	109	6	6	121												
Coredo	Val di Non	human	247	30	34	311	2			0.81 (0.1–2.9)		0.6 (0.08–2.3)						
Sores	Val di Non	human	85	9	10	104			1		5.3 (0.1–26)	1.0 (0.02–5.2)	0.17 (0.02–0.6)	0.48 (0.01–2.6)	0.21 (0.04–0.6)			
Vervo	Val di Non	human	30	10	7	47												
Brez	Val di Non	human	286	10	8	304												
Arsio	Val di Non	human	89	23	19	131												
Fondo	Val di Non	human	79	8	3	90										0.10 (0.01–0.36)	0.53 (0.06–1.9)	0.17 (0.05–0.42)
Livo	Val di Non	human	164	9	4	177												
Filari	Valle dei Laghi	human	237	2	5	244												
Lamar	Valle dei Laghi	human	139	21	20	180												
Calisio	Val d'Adige	human	138	35	55	228			1		1.1 (0.03–6)	0.4 (0.01–2.4)						
Ronchi	Valsugana	goat	23	0	0	23												
Nomeson	Vallagarina	goat	135	12	16	163												
Bivedo	Valli Giudicarie	goat	149	2	0	151												
Predazzo	Val di Fiemme	goat	13	0	0	13												
			2032	185	193	2410												

*N* = nymphs; *A* = adults. The 95% confidence intervals of TBEV prevalence are reported in brackets.

Questing ticks were collected during the period May-September 2018 by dragging a standard 1 m^2^ white blanket for about one hour at each site above lower vegetation along tracks and ecotones of deciduous and mixed forest. All ticks were identified microscopically by species, sex and life stage using taxonomic keys [27]. Larvae were discarded. All individuals identified as *I. ricinus* were washed once in 70% ethanol followed by deionized water for 5 min, divided according to stage, locality and date of collection, and then stored at −80°C until nucleic acid extraction. Adults were treated individually, while nymphs were grouped in pools of 10 when possible, with few pools ranging from 3 to 11 specimens depending on the number of ticks collected from a given locality (pool size average = 9.7; standard deviation = 0.8).

### RNA extraction

Before RNA isolation, ticks were thawed on ice. Individual adults or nymph pools were homogenized in 350 μl buffer RLT with 5 mm stainless steel beads (Qiagen) in a MM200 mixer mill (Retsch) for 4 min at 25 s^−1^. Total RNA was extracted from homogenized ticks using the RNeasy Mini Kit (Qiagen), quantified with NanoDrop 8000 Spectrophotometer (Thermo Fisher Scientific) and Qubit 2.0 Fluorometer (Thermo Fisher Scientific), and stored at −80°C.

### Real-time RT–PCR for TBEV detection

Samples were screened for the presence of specific TBEV RNA by a real-time Taq-Man based one-step RT–PCR targeting a fragment of the 3’ non-coding region of TBEV genome as described previously [28]. Extracted RNA of strain Hypr was used as a positive control, while distilled water served as a negative control. Each reaction, either on tick samples or controls, was performed in duplicate in a 7300 Real Time PCR system (Applied Biosystems).

### RT–PCR and sequencing of the E gene

Samples positive by real-time PCR were confirmed by a one-step RT–PCR which amplifies a fragment of the E gene (about 520 bp long) as described previously [29]. After being visualized via capillary electrophoresis on a QIAxcel system (Qiagen), the PCR products were purified using the MinElute PCR Purification Kit (Qiagen) and sequenced on an ABI 3730xl DNA Analyzer (Applied Biosystems).

### Phylogenetic analysis

The partial E gene sequences generated by RT–PCR were trimmed off the primers and aligned with complete or partial E gene sequences belonging to 67 TBEV strains retrieved from GenBank. Strains from twenty European countries, Japan, South Korea and China were included. The Italian TBEV sequences from GenBank included 3 sequences identified in humans in Friuli Venezia Giulia (FVG) in 2013 and 2 in ticks in the Province of Trento in 2006 [22]. The Omsk haemorrhagic fever virus (OHFV) was used as an outgroup. Phylogenetic analysis was performed using the maximum-likelihood method based on the general time reversible substitution model with among-site rate heterogeneity modelled by the Γ distribution available in RAxML v8 [30], including 1,000 bootstrap replicates to determine the node support. Provean [31], PredictSNP [32] and SIFT [33] were used to predict the effect of the amino acid changes on TBEV E protein function due to the non-synonymous substitutions observed among our sequences and the reference Neudoerfl strain.

### Statistical analysis

The infection prevalence was calculated using the minimum infection rate (MIR): (p/N)x100%, where *p* = the number of positive pools or individuals and *N* = the total number of ticks tested. MIR is based on the assumption that only one infected tick is present in each positive pool [34], which is acceptable for arboviruses occurring at low prevalences in their vector populations, such as TBEV. The confidence interval of MIR was calculated using the binomial Clopper–Pearson “exact” method using the online EpiTools epidemiological calculator (http://epitools.ausvet.com.au). The significance of the differences in infection prevalence between tick developmental stages and sampling sites were evaluated using the Fisher’s exact test.

## Results

### Human TBE cases

Between 1992 and 2019, 174 human TBE cases were diagnosed in the Province of Trento, Italy. Seventy-one were reported in Val di Non, whereas 33, 24, 21 and 10 cases were documented in Valle dei Laghi, Val di Cembra, Val d’Adige and Vallagarina, respectively (Suppl. Tab. 1; Fig. 1). In the period 1992–1999, only ten cases were reported, exclusively from Valle dei Laghi. From 2000 to 2011, on a total of 43 cases, 27 were recorded in other valleys as well, mainly in Val di Non (15), but still at a relatively low incidence (average IR: 0.73 per 100,000 inhabitants). Since 2012 to present an upsurge in TBE incidence was observed (average IR: 2.82; 121 cases), with peaks in 2017 and 2019, when 19 (IR: 3.53) and 25 (IR: 4.62) human TBE infections were diagnosed in the Province respectively (Suppl. Tab. 1; Fig. 2). In 2017, most cases (13, i.e. 68%) were recorded in Val di Non, 7 of which were concentrated in 4 hamlets within the municipality of Predaia, while 3 were reported in the provincial capital (Trento) or in its immediate surroundings (Lavis) (Suppl. Tab. 2; Fig. 1).

### Serological investigation

Serological screening of all the 706 goat serum samples by ELISA yielded 9 (1.3%) positive and 107 (15.1%) borderline results. The subsequent VNT performed on these 116 samples yielded 11 positive samples, 6 of the 9 ELISA positive and 5 of the 107 ELISA borderline samples, belonging to 5 different farms (Fig. 1; Suppl. Tab. 3). The overall seroprevalence in the province of Trento was 1.56%.

### TBEV in questing ticks

A total of 2,410 questing ticks were collected: 2,032 (84.3%) were nymphs and 378 (15.7%) adults, of which 185 (48.9%) males and 193 (51.1.%) females (Table 1). All ticks belonged to the species *I. ricinus*. The majority (58.4%) of ticks were collected in Val di Non, where most of the human cases were reported in 2017 (68%). Two females and 2 pools of 10 nymphs each resulted positive by real-time RT–PCR. The 2 nymph pools and one female were collected in Val di Non, in the localities Coredo (891 m a.s.l.) (“CORN10” and “CORN12” strains) and Sores (1,109 m a.s.l.) (“SORF3”) respectively, both belonging to the municipality of Predaia (Table 1). The other positive female was collected on Mount Calisio (833 m a.s.l.) (“CALF15”), municipality of Trento, Val d’Adige (Table 1). The TBEV prevalence in ticks by site, valley and life stage is reported in Table 1. The overall prevalence in the study area was 0.17% [95% CI: 0.05–0.42%], ranging between 0.53% [95% CI: 0.06–1.90%] in adults and 0.10% [95% CI: 0.01–0.36%] in nymphs. The differences in prevalence between adults and nymphs, valleys and sites were not statistically significant (*p* = 0.11; *p* = 0.45–1; *p* = 0.53–1, respectively).

### E gene sequencing

**Figure 3.**
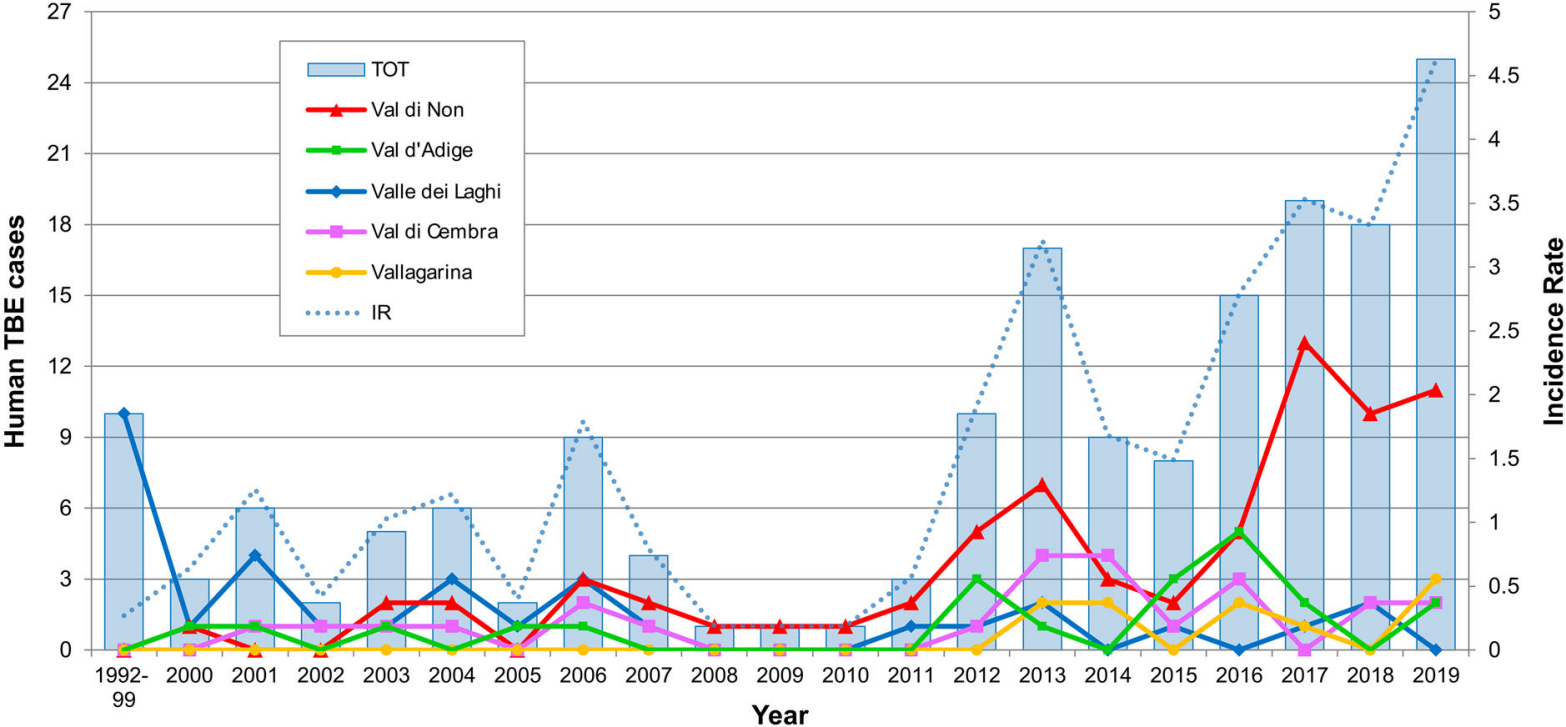
Variable sites in the E protein gene sequences between the TBEV strains from this study and the reference Neudoerfl strain. Nucleotide (upper) and amino acid (lower) positions are related to the complete polyprotein sequence. Nucleotide and amino acid substitutions are highlighted by colours.

A 520 bp long fragment of the E gene was amplified by RT–PCR and sequenced by Sanger sequencing for each of the 4 TBEV positive samples, except CORN12 for which a shorter fragment (460 bp) was obtained. Each sequence was compared for identity against the complete NCBI nucleotide database by BLAST search, revealing the highest similarity with strains belonging to TBEV-Eu. In particular, CORN10, CORN12 and SORF3 were identical to each other and showed 98.5% nucleotide similarity (8 nucleotide substitutions, 7 for CORN12) to the reference strain of the European subtype (Neudoerfl, accession no. U27495) (Figure 3). In contrast, CALF15 differed from the other strains by 3 nucleotide substitutions (99.4% identity), and from the Neudoerfl strain by 11 (97.9% identity) (Figure 3). Only 2 of the observed substitutions were non-synonymous: Thr→Ile at aa 408 between all the strains, including Neudoerfl, and CALF15; Val→Ile at aa 447 between all the strains and Neudoerfl (Figure 3). Both mutations were predicted to be neutral on TBEV E protein function based on Provean (score −1.8 and −0.59, respectively), SIFT (score 0.92 and 0.98, respectively) and PredictSNP (score 65% for both) results.

### Phylogenetic analysis

**Figure 4.**
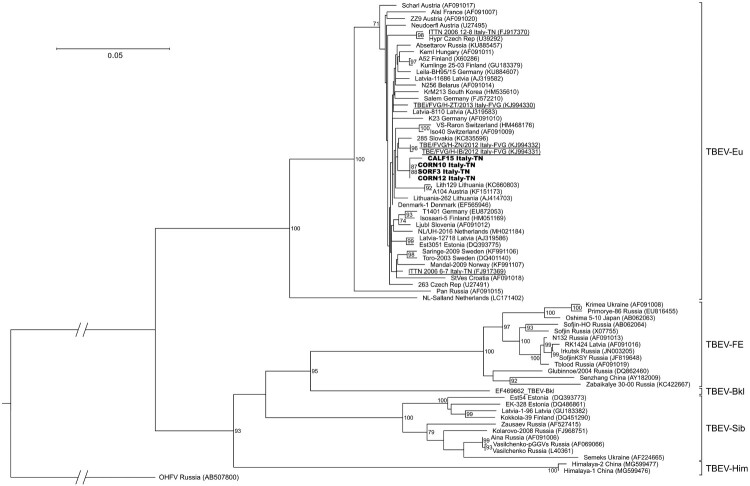
Phylogenetic tree of the partial E gene sequences generated in this study together with 67 TBEV strains retrieved from GenBank. Sequences obtained in this study are shown in bold, while those identified in the Province of Trento in 2006 (Italy-TN) and in Friuli Venezia Giulia in 2013 (Italy-FVG) are underlined. Only bootstrap values exceeding 70% are shown. The scale bar indicates 0.05 nucleotide substitutions per site.

All sequences clearly clustered in 2 main clades: one included TBEV-Eu, while TBEV-FE, TBEV-Sib, TBEV-Bkl and TBEV-Him were grouped together, with TBEV-FE and TBEV-Sib representing sister groups and TBEV-Him forming a separate lineage (Figure 4). Based on the bootstrap values, most basal nodes were highly supported. The sequences of the 4 strains from the present study were placed inside the clade of TBEV-Eu, where they clustered together in one group and were close to the sequences obtained from FVG and from neighbouring countries, such as Austria, Switzerland and Slovakia. In contrast, the other sequence from FVG and the 2 sequences identified in the Province of Trento in 2006 clustered further apart. The sequences within the TBEV-Eu clade demonstrated a high level of nucleotide similarity (96–100%), excluding the Salland and Pan strains (6% and 10% divergence from the other strains, respectively). The TBEV-Eu strains did not show any geographical clustering, and the terminal nodes had low support and were not always completely resolved.

## Discussion

Between 2012 and 2019 a sharp increase in TBE incidence was reported in the Province of Trento, with an average IR (2.82) almost 4 times higher than previous years (2000–2011) (0.73) (Figure 2). Such value is also higher than the IR of 0.52 registered in northeast Italy in the same time frame [35]. This trend is consistent with the general increase in the number of human TBE cases in endemic regions of Europe, where TBE risk areas have spread northwards and to higher altitudes, and new foci have been discovered [36,37]. In addition, more than 70% of the territory of Province of Trento is located over 1,000 m a.s.l. and 55% of its surface is covered by coniferous and deciduous forests, which represent a favourable habitat to the occurrence of tick vectors and wildlife species playing key roles in TBEV transmission, such as rodents and ungulates [20]. Furthermore, the popularity in the area of outdoor recreational activities, such as hiking, camping and mushroom and berry picking, which favour human exposure to ticks, represents an important local risk factor for TBE infection [16,18].

In the Province of Trento vaccination for TBE has been offered by public health authorities only to professionally exposed groups until January 2018, since when, due the increase in the number of cases, the TBE vaccine (TICOVAC, Pfizer) has been offered free of charge to the whole resident population. Nevertheless, the number of cases remained high in 2019.

As solicited by the increase in human TBE cases, especially in 2017 when a peak in incidence of 3.53 was observed (Figure 2), we applied an integrated approach combining multiple methods to identify active TBE foci in the Province of Trento and assess spatial variation in TBE hazard in respect to what was observed in past studies [19,21,22]. In particular, we used the TBEV seroprevalence in vertebrate sentinel hosts (goats) and the spatio-temporal distribution of human TBE cases to concentrate the screening of questing ticks for TBEV in areas with evidence of TBEV circulation. This is the first case that such an integrated approach is applied on a vast area, including several valleys, of this territory, where TBE hazard has been always evaluated using one single method and focusing on limited areas [19,21,22].

Such an integrated approach could better overcome the limitations of each one of the above mentioned methodologies. Indeed, human surveillance alone is not a reliable indicator since it may be influenced by vaccination coverage, exposure levels and public awareness of TBE risk. Detection of TBEV in ticks, although providing a direct evidence for the presence of the virus in a given area, is usually time-consuming and expensive, since tick distribution is overdispersed in the environment and TBEV generally occurs in ticks at low prevalence (0.1–5% in Europe) with a patchy distribution in small foci [6,7], thus requiring large sample numbers and a proper selection of the target area. Finally, detection of TBEV-specific antibodies in sentinel vertebrate hosts is faster and cheaper, but is less informative about time and place of infection since TBEV produces long-term immunity in its hosts [11]. Goats, in particular, represent suitable sentinels for TBE, and have been often used for this purpose [11,19,24,38], since they are sedentary and are usually kept in small and defined pastures for long periods of time, where they can be repeatedly bitten by ticks and get eventually infected with TBEV.

TBEV detection in ticks confirmed the emergence of two TBE foci in the Province of Trento. One was identified in Val di Non, where 3 of the 4 tick samples positive to TBEV during our screening were collected. Here, despite human cases have been regularly recorded every year since 2006, a dramatic increase in TBE incidence was observed since 2017 (Figure 2), which seems to indicate the emergence (intensification) of an already existing focus. In addition, the detection of TBEV at an altitude (Sores: 1,109 m a.s.l.) considerably higher than in previous reports of TBEV in this area (721 m a.s.l.) [22] is consistent with the general expansion of TBE at increasing altitudes observed in central Europe in the last decade [39,40], most likely as a consequence of climate warming driving the dispersal of ticks at higher elevations. Another focus was found in Val d’Adige, where an increase in human cases was documented between 2015 and 2017 (Suppl. Tab. 1), and, in particular, in proximity of Trento, the provincial capital. Indeed, 3 human cases were registered in Trento or in its immediate surroundings in 2017 and TBEV was detected in a tick collected on a mountain (Mt. Calisio) located right above the city. This TBEV focus may represent a relevant risk factor for the inhabitants of the city which commonly use this area for recreational outdoor activities. On the opposite, we did not find infected ticks in the previously known TBE focus in Valle dei Laghi, where most human cases were reported between 1992 and 2006 (24 cases), and where TBEV was detected in ticks in 1996–97 [21] and 2006 [22]. Since human cases are still recorded in this valley, even if at lower frequency than in the past (9 cases since 2007), we hypothesize that TBEV circulation is still maintained here, but at very low level, possibly due to variation in local ecological drivers (hosts, climate), which need to be investigated. In addition, local people may have become more aware of TBE risk, due to the historical presence of TBE in this valley.

Our results revealed an overall good correspondence between human TBE cases and TBEV detection in ticks, which contrasts with the general idea that TBEV prevalence in ticks is not a sensitive indicator of infection risk to humans [8,41]. We demonstrated that TBEV detection in ticks can be successfully used to confirm the identification of TBEV foci in areas where other evidences suggest the circulation of TBEV, thus highlighting the importance of an integrated approach in assessing TBE hazard.

None of the five hypothetical TBE risk areas suggested by the serological screening on goats was confirmed by TBEV detection in ticks. Four of them do not have historical records of human TBE cases, which is in contrast with the usually good spatial correlation between TBE incidence in humans and seroprevalence in bovids [8], while one was located in Vallagarina, where human TBE cases have been reported since 2013 (Fig. 1; Suppl. Tab. 1). These results may reveal the emergence of new TBEV foci where human cases have not yet occurred or have been misdiagnosed. Unfortunately, very few ticks were collected from these sites, which could partly explain why we did not find infected ticks. Further screenings of ticks should be performed in these areas to confirm the circulation of TBEV.

The overall estimated TBEV prevalence in ticks was 0.17% in the Province of Trento, with values of 0.21% in Val di Non and 0.4% in Val d’Adige. Our findings reveal an increase in TBEV prevalence in this area compared to what observed in 1996–1997 [21], and are in accordance with the estimate registered in 2005–2006 in the adjacent TBE endemic region of Friuli Venezia Giulia (0.21%) [42]. The higher estimates reported from the Province of Trento in 2006 [22] (2.5%) and from northeast Italy (Friuli Venezia Giulia and Veneto) in 2006–2008 [43] (2.1%) can be explained by the fact that only adult ticks were analyzed in these studies, resulting in a partial assessment of TBEV prevalence. Furthermore, while Carpi et al.[22] targeted a single known focus, we included in our study a much larger area of the Province, likely sampling ticks also in non-endemic areas adjacent to the TBEV foci, and thus resulting in lower prevalence estimates. Our estimates are within the range of TBEV prevalence registered in European endemic areas (0.1–5%) [6,7], and similar to the estimates reported in neighbouring countries, such as Switzerland (0.10–0.46%) [38,44–46], Slovenia (0.30–0.47%) [10,47], Germany (0.10–0.60%) [7,48–50] and France (0.10–0.24%) [51,52].

The phylogenetic analysis demonstrated that the TBEV strain circulating in the Province of Trento belongs to the TBEV-Eu subtype (Figure 4), consistently with the results of previous studies carried out in this area [21,22,42]. The TBEV strains clustered in function of the tick vector species: the TBEV-Eu strains, which are primarily vectored by *I. ricinus*, formed one clade, while the eastern strains (TBEV-FE, TBEV-Sib and TBEV-Bkl), mainly carried by *I. persulcatus*, and TBEV-Him, whose tick vector is still unknown, grouped together (Figure 4). All the Italian sequences produced so far were scattered across the TBEV-Eu clade and, in particular, our sequences clustered all together but distantly from those generated in the Province of Trento in 2006 [22] and, partially, also from those of Friuli Venezia Giulia (Figure 4). This seems to indicate a distinct and independent origin of these strains, which may have originated from different introductions from neighbouring countries through hosts movements, e.g. migratory birds. The carriage of TBEV or TBEV-infected ticks by birds is thought to be implied in the recent spread of TBEV-Eu strains throughout Europe, which explains the absence of geographical clustering and the low genetic variability within the TBEV-Eu clade [53,54]. Our analysis of a fragment of the E gene confirmed the generally low genetic variability between the TBEV-Eu strains (2.5%). Of the only two non-synonymous substitutions observed among our sequences and the reference Neudoerfl strain (Figure 3), the amino acid T408I substitution observed in CALF15 may be interesting since this position is highly conserved as a Threonine among mammalian tick-borne flaviviruses, with the exception of the Powassan virus only. Even if Threonine and Isoleucine differ in hydropathy, this substitution seems to have a neutral effect on the function of TBEV E protein, according to the prediction tools used. Nevertheless, the role of this residue in TBEV E protein is as yet unknow and future research is needed to investigate the effect of this substitution. The I447 V substitution between the Neudoerfl and our strains is highly conservative, considering the common physical properties of the amino acids involved and that a Valine is observed in all TBEV strains, except Neudoerfl.

In conclusion, our study provides an updated picture of TBE hazard in the Province of Trento demonstrating an increase in TBEV prevalence in ticks more than a decade after previous reports and the presence of anti-TBEV antibodies in goats, and suggesting the emergence of two active TBEV foci in the area. Our study highlights the importance of an integrated approach for TBE risk assessment in order to overcome the limits of each surveillance method, and that TBEV detection in ticks can be successfully used to confirm the circulation of TBEV in areas suggested by other evidences, here human cases. Future efforts should focus on elucidating the variation in the ecological factors which have led to the emergence of the foci here identified. Our data point out the importance of maintaining the TBE vaccination policy recently adopted in the Province of Trento. However, we believe that more efforts should be concentrated in public health education in order to increase the awareness of TBE infection risk, given the popularity of outdoor recreational activities among residents and tourists in this region.

## Supplementary Material

Supplemental Material

## Data Availability

The partial TBEV E gene fragment sequences generated in this study were deposited in GenBank under the accession number MN746771-4.
