# Correction

**DOI:** 10.1080/22221751.2020.1737359

**Published:** 2020-03-08

**Article title:** Tick-borne encephalitis foci in northeast Italy revealed by combined virus detection in ticks, serosurvey on goats and human cases

**Authors:** Alfano, N., Tagliapietra, V., Rosso, F., Ziegler U., Arnoldi D., & Rizzoli, A.

**Journal:** *Emerging Microbes & Infections*

**Bibliometrics:** Volume 9, Number 1, pages 474–484

**DOI:** https://doi.org/10.1080/22221751.2020.1730246

When the article was first published online, there were mistakes in Figures 2 and 3 and their text citations. These have now been corrected in online version.

