# Supplementary material for: Tick-borne encephalitis foci in northeast Italy revealed by combined virus detection in ticks, serosurvey on goats and human cases
Source: Emerg Microbes Infect. 2020 Feb 26;9(1):474–84. doi: 10.1080/22221751.2020.1730246 (PMC7054962; doi:10.1080/22221751.2020.1730246)
Supplement: Supplemental Material [file TEMI_A_1730246_SM0494.zip › Supplementary material.docx]

**Suppl. Tab. 1.** Temporal and spatial distribution by valley of human TBE cases in the Province of Trento. IR ^a^: the incidence rate is calculated as the number of cases/resident population per 100.000 inhabitants.

**Suppl. Tab. 2.** Human TBE cases by locality, municipality and valley registered in the Province of Trento in 2017.

**Suppl. Tab. 3.** Results of the serological screening on goats with location of the farms tested in the Province of Trento.
